# The Epidemiology of Neuroendocrine Tumors in Taiwan: A Nation-Wide Cancer Registry-Based Study

**DOI:** 10.1371/journal.pone.0062487

**Published:** 2013-04-22

**Authors:** Hui-Jen Tsai, Chun-Chieh Wu, Chia-Rung Tsai, Sheng-Fung Lin, Li-Tzong Chen, Jeffrey S. Chang

**Affiliations:** 1 National Institute of Cancer Research, National Health Research Institutes, Tainan, Taiwan; 2 Department of Internal Medicine, National Cheng Kung University Hospital, Tainan, Taiwan; 3 Department of Internal Medicine, Kaohsiung Medical University Hospital, Kaohsiung Medical University Hospital, Kaohsiung, Taiwan; 4 Graduate Institute of Medicine, College of Medicine, Kaohsiung Medical University, Kaohsiung, Taiwan; 5 Department of Pathology, Kaohsiung Medical University Hospital, Kaohsiung, Taiwan; 6 Institute of Molecular Medicine, National Cheng Kung University, Tainan, Taiwan; The University of Texas M. D. Anderson Cancer Center, United States of America

## Abstract

**Background:**

The epidemiology of neuroendocrine tumors (NETs) is not well illustrated, particularly for Asian countries.

**Methods:**

The age-standardized incidence rates and observed survival rates of NETs diagnosed in Taiwan from January 1, 1996 to December 31, 2008 were calculated using data of the Taiwan Cancer Registry (TCR) and compared to those of the Norwegian Registry of Cancer (NRC) and the US Surveillance, Epidemiology, and End Results (SEER) program.

**Results:**

During the study period, a total of 2,187 NET cases were diagnosed in Taiwan, with 62% males and a mean age of 57.9 years-old. The age-standardized incidence rate of NETs increased from 0.30 per 100,000 in 1996 to 1.51 per 100,000 in 2008. The most common primary sites were rectum (25.4%), lung and bronchus (20%) and stomach (7.4%). The 5-year observed survival was 50.4% for all NETs (43.4% for men and 61.8% for women, *P*<0.0001). The best 5-year observed survivals for NETs by sites were rectum (80.9%), appendix (75.7%), and breast (64.8%).

**Conclusions:**

Compared to the data of Norway and the US, the age-standardized incidence rate of NETs in Taiwan is lower and the major primary sites are different, whereas the long-term outcome is similar. More studies on the pathogenesis of NETs are warranted to devise preventive strategies and improve treatment outcomes for NETs.

## Introduction

Neuroendocrine tumors (NETs) are neoplasms originating from neuroendocrine cells located throughout the body, most commonly in lung and gastrointestinal tract [1], [2]. NETs may secrete various peptides, some of which may cause clinical symptoms (also known as “functioning” NET) [3]. Most NETs have an indolent course, whereas some proliferate rapidly and metastasize to distant organs. Due to their heterogeneity, the first World Healthcare Organization (WHO) classification of NETs was not established until 1980. In 2000, the WHO classification of NETs was updated based on histopathology and was revised again in 2010 [4]. The incidence rate of NETs was not well-known until recently when Yao et al. and Hauso et al. published their surveys of NETs using data from the US Surveillance, Epidemiology, and End Results (SEER) program and from the Norwegian Registry of Cancer (NRC) [1], [2]. The NETs incidence rate was 1.09 per 100,000 in 1973 and increased to 3.31 and 5.25 per 100,000 in 1993 and 2004, respectively, based on the SEER data [2]. The NETs incidence rate in Norway increased from 2.35 per 100,000 during 1993–1997 to 4.06 per 100,000 during 2000–2004 [1]. In the US, Asian/Pacific Islanders had a lower incidence rate of NETs (3.19 per 100,000) than Whites (4.92 per 100,000) and African Americans (6.82 per 100,000). [2] A large-scaled epidemiological survey of gatroenteropancreatic (GEP)-NETs (n = 2,845) in Japan estimated that the annual incidence rate of GEP-NETs was 1.01 per 100,000 [5], which was lower than those in the US (2.85 per 100,000) and Norway (2.33 per 100,000). These results suggested a racial disparity in the incidence rate of NETs. However, there has been a paucity of data to comprehensively describe the epidemiology of NETs among Asians in Asia. This study analyzed the incidence rate and the observed survival rate of NETs in Taiwan by using data from the Taiwan Cancer Registry (TCR) from 1996 to 2008 and compared them to the NRC and the SEER data. To our knowledge, this is the first nation-wide cancer registry-based study of NETs from Asia.

## Materials and Methods

The incident NET cases diagnosed between January 1, 1996 and December 31, 2008 were identified from the TCR, which was established in 1979 to monitor the incidence and the mortality rates of cancer in Taiwan [6]. Hospitals with 50 or more beds in Taiwan are required to report cancer diagnoses to the TCR and the number of reporting hospitals increased from 179 in 1996 to 205 in 2008. Although some cases of cancer may be initially suspected by doctors in the clinics, the diagnosis of cancer is ultimately confirmed by the hospitals. Under the current system, the TCR captures 97% of the cancer cases in Taiwan [6]. The quality of a cancer registry is indicated by the percentage of death certificate only cases (DCO%) and the percentage of morphologically verified cases (MV%), with the perfect data quality represented by a DCO% of 0 and a MV% of 100 [7]. The DCO% of the cancer cases in the TCR decreased from 14.2% in 1996 to 1.2% in 2008 [6]. The MV% ranged from 87.5% in 2002 to 89% in 2008 [6]. These indices indicate that the quality of the TCR is comparable to the other well-established cancer registries in the world [8], [9]. For the NRC, 2001–2005, the DCO% was 0.9% and the MV% was 93.8% [8]. For the US SEER program, 1998–2002, the DCO% was 1.0% and the MV% was 94.7% [9].

The morphology (M) codes of the International Classification of Diseases for Oncology, Field Trial Edition (ICD-O-FT) (for those diagnosed from January 1, 1996 to December 31, 2001) or the International Classification of Diseases for Oncology, Third Edition (ICD-O-3) (for those diagnosed after January 1, 2002) were used to identify NET cases. We adopted the same M codes used by Hauso et al [1]. The M codes for NETs were: 8240 (carcinoid tumor), 8241 (enterochromaffin cell carcinoid), 8242 (enterochromaffin-like cell tumors), 8243 (goblet cell carcinoid), 8244 (composite carcinoid), 8245 (adenocarcinoid), 8246 (neuroendocrine carcinoma). Three M codes of NETs appear only in ICO-O-3∶8249 (atypical carcinoid), 8013 (large cell neuroendocrine carcinoma), and 8574 (adenocarcinoma with neuroendocrine differentiation). The ICD codes to identify the sites of NETs are presented in Table S1.

The crude annual incidence rates of NETs in Taiwan from 1996 to 2008 were calculated for all sites combined, by each site, and by sex, using the annual population reported by the Directorate-General of Budget, Accounting, and Statistics of Taiwan (http://www.dgbas.gov.tw). To compare with data reported by Hauso et al. [1] and Yao et al. [2], all incidence rates were age-standardized using the 2000 US standard population, which was also used by Hauso et al. and Yao et al. to calculate the age-standardized incidence rates. In addition, the male to female (M/F) case number ratios for all NETs and by sites were calculated. The M/F case number ratio of NETs at each site was compared to the M/F case number ratio of the most common histologic tumor type at the same site (adenocarcinoma (AC) for rectum, lung and bronchus, stomach, pancreas, colon, and small intestine; squamous cell carcinoma (SCC) for lung and bronchus and head and neck) in order to evaluate whether NETs might share common risk factors with tumors of other histologic types occurring at the same sites. The M/F case number ratios of AC and SCC were calculated using the incident case numbers reported by the TCR [6]. The distribution of sex by histologic types at each site was evaluated by chi-square test.

The date of death for the NET cases was determined by linking the TCR data to the national death database. The life-table method was used to calculate the 5-year observed overall survival (OS) of NETs for all sites combined, by each site, and by sex. Cox proportional hazards regression model was performed to estimate the hazard ratio (HR) and 95% confidence interval (CI) of NET death associated with body site, age, and sex. Because the TCR has incomplete information on the stage and grade of NETs, these two factors were excluded from the survival analysis. This study was approved by the Institutional Review Board of the National Health Research Institutes.

## Results

### Age-Standardized Incidence Rates

**Figure 1:**
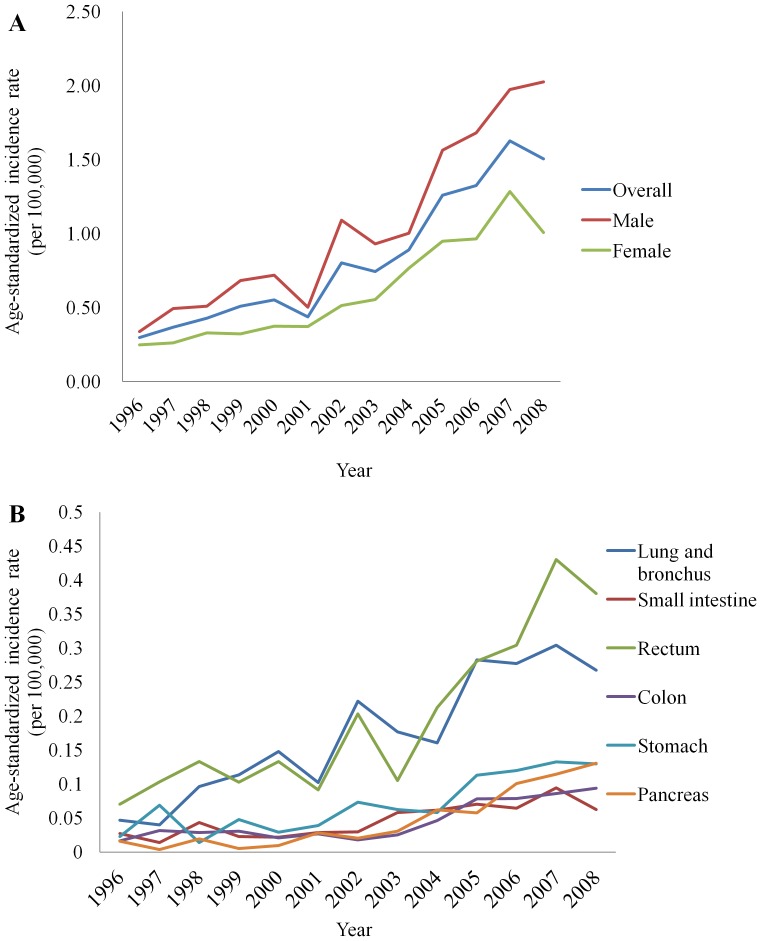
The age-standardized incidence rate of neuroendocrine tumors, Taiwan, 1996–2008. A) The age-standardized incidence rate overall and by sex; B) The age-standardized incidence rate by primary sites.

A total of 2,187 newly diagnosed NET cases were recorded in the TCR from January 1, 1996 to December 31, 2008 with 1,356 (62%) men and a mean age of 57.9 years-old (range: 9–95; 70% diagnosed at ≧ 50 years-old). Because the WHO classification of NETs was updated based on histopathology in 2000 [4], we examined the change in the incidence rate of NETs before and after 2000. The age-standardized annual incidence rate of NETs in Taiwan increased from 0.30 per 100,000 in 1996, to 0.55 per 100,000 in 2000, and to 1.51 per 100,000 in 2008 (Figure 1A and Table 1). The age-standardized incidence rate of NETs increased by 83% from 1996 to 2000 and by 175% from 2000 to 2008. Men consistently had a higher incidence rate of NETs than women and the male to female incidence rate ratio increased from 1.4 in 1996 to 2.0 in 2008. All of the six most common NETs by sites (rectum, lung and bronchus, stomach, pancreas, colon, and small intestine) experienced a rise in the incidence rate from 1996 to 2008 (Figure 1B and Table 1).

**Table 1:** Age-standardized incidence rate (per 100,000) of neuroendocrine tumors, Taiwan, 1996–2008.

	Age-standardized incidence rate per 100,000^a^								
Year	All cases	Sex		Sites					
		Male	Female	Rectum	Lung & bronchus	Stomach	Pancreas	Colon	Small intestine
1996	0.30	0.34	0.25	0.07	0.05	0.02	0.02	0.02	0.03
1997	0.37	0.49	0.26	0.10	0.04	0.07	0.004	0.03	0.01
1998	0.43	0.51	0.33	0.13	0.10	0.01	0.02	0.03	0.04
1999	0.51	0.68	0.32	0.10	0.11	0.05	0.005	0.03	0.02
2000	0.55	0.72	0.38	0.13	0.15	0.03	0.01	0.02	0.02
2001	0.44	0.50	0.37	0.09	0.10	0.04	0.03	0.03	0.03
2002	0.80	1.09	0.52	0.20	0.22	0.07	0.02	0.02	0.03
2003	0.74	0.93	0.55	0.11	0.18	0.06	0.03	0.03	0.06
2004	0.89	1.00	0.77	0.21	0.16	0.06	0.06	0.05	0.06
2005	1.26	1.56	0.95	0.28	0.28	0.11	0.06	0.08	0.07
2006	1.32	1.68	0.96	0.30	0.28	0.12	0.10	0.08	0.07
2007	1.63	1.97	1.28	0.43	0.30	0.13	0.11	0.09	0.09
2008	1.51	2.03	1.01	0.38	0.27	0.13	0.13	0.09	0.06

a Incidence rates were age-standardized to the 2000 US standard population.

### Distributions of NETs by Sites and Sex

**Figure 2:**
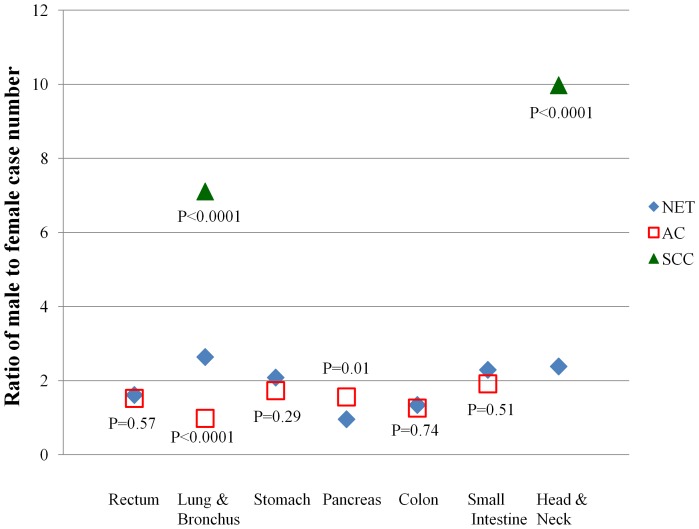
The comparison of male to female case number ratios by histologic subtypes in different primary sites, Taiwan. Abbreviations: AC = adenocarcinoma; NET = neuroendocrine tumor; SCC = squamous cell carcinoma. P-values were generated by chi-square tests to compare the distribution of sex between AC or SCC and NET.

The most common primary sites of NETs were rectum followed by lung and bronchus, stomach, pancreas, colon, and small intestine (Table 2). For both men and women, the most common primary sites of NETs were rectum followed by lung and bronchus (Table 2). Excluding those arising from sex-specific organs, the M/F case number ratios of primary NETs involving upper aero-digestive tracts (head and neck, lung and bronchus and esophagus), stomach, and small intestine, were >2. The M/F case number ratios of primary NETs arising from appendix, colon, rectum, and liver ranged from 1 to 2, whereas the M/F case number ratios were <1 for primary NETs of the biliary tract (gallbladder and extrahepatic bile duct) and pancreas. Compared to the M/F case number ratios of the most common cancer subtypes by primary sites, the M/F case number ratio of NETs appeared similar (*P*>0.05) to the M/F case number ratio of AC in rectum, stomach, colon, and small intestine, but was higher (*P*<0.0001) than the M/F case number ratio of AC in lung and lower (*P = *0.01) than the M/F ratio of AC in pancreas. The M/F case number ratio of NETs was lower (*P*<0.0001) than that of SCC in lung or head and neck (Figure 2).

**Table 2:** Distribution of neuroendocrine tumors by sites, Taiwan, 1996–2008.

	All Cases		Male		Female		Male to female case number ratio
	*N* = 2,187		*N* = 1,356		*N* = 831		
Sites	n	%	n	%	n	%	
Rectum	555	25.4	342	25.2	213	25.6	1.61
Lung and bronchus	437	20.0	317	23.4	120	14.4	2.64
Stomach	163	7.4	110	8.1	53	6.4	2.08
Pancreas	131	6.0	64	4.7	67	8.1	0.96
Colon	117	5.3	67	4.9	50	6.0	1.34
Small intestine	115	5.3	80	5.9	35	4.2	2.29
Head and neck^a^	88	4.0	62	4.6	26	3.1	2.38
Appendix	78	3.6	49	3.6	29	3.5	1.69
Liver	37	1.7	21	1.6	16	1.9	1.31
Breast	34	1.5	1	0.1	33	4.0	0.03
Esophagus	22	1.0	21	1.6	1	0.1	21
Ovary	20	0.9	–	–	20	2.4	–
Prostate	12	0.6	12	0.9	–	–	–
Biliary^a^	12	0.6	4	0.3	8	1.0	0.5
Others^a^	366	16.7	206	15.2	160	19.3	1.29

a. Head and neck includes lip and oral cavity, pharynx, larynx, nasal cavity and paranasal sinuses, middle ear, and major salivary glands; Biliary includes gallbladder and extrahepatic bile duct; Others includes anus, bone, brain, cervix, intracranial gland, kidney, labia majora, mediastinum of the heart, peritoneum, pleura, retroperitoneum, skin, testis, thymus, thyroid, urinary bladder, uterus, vagina, and site undefined.

### Survival

The 5-year observed survival was 50.4% for all NETs (43.4% for men and 61.8% for women, *P*<0.0001) (Table 3). Patients with rectal NETs experienced the best survival with a 5-year observed survival of 80.9%, followed by NETs of the appendix (75.7%) and the breast (64.8%). Patients with esophageal NET had the worst prognosis with a 5-year observed survival of 14.3%. Among NETs in men, the best 5-year observed survivals were 77.5%, 76.8%, and 50.0% for NETs in rectum, appendix and biliary tract (gallbladder and extrahepatic bile duct), respectively. For women, the best 5-year observed survivals were 86.4%, 73.8%, and 64.7% for NETs in rectum, appendix, and stomach, respectively. The prognosis of NETs for women was better than men for all sites except for NETs in the biliary tract. NETs in the other sites had a higher HR of death (*P*<0.05) compared to rectal NET in the univariable analysis, except for appendix (HR = 1.38, 95% CI: 0.87–2.18) and ovary (HR = 1.84, 95% CI: 0.86–3.96) (Table 4). In the multivariable analysis (Table 4) adjusted for sex and age, the risk of death for NETs in the appendix was still not different from that of rectal NET and NETs in the other sites had a worse prognosis than rectal NET. Being female and age<50 were independent favorable prognostic factors for the OS of NETs.

**Table 3:** 5-year observed survival probability of neuroendocrine tumors, Taiwan, 1996–2008.

	5-Year observed survival probability (%)^a^				
Site		Overall	Male	Female	*P* for sex difference^b^
All site		50.4	43.4	61.8	<0.0001
Rectum		80.9	77.5	86.4	0.009
Lung and bronchus		33.9	22.8	63.4	<0.0001
Stomach		46.4	37.6	64.7	0.0004
Pancreas		30.2	16.3	45.8	0.02
Colon		48.1	45.0	52.5	0.40
Small intestine		47.9	42.8	58.8	0.23
Head and neck^c^		48.0	40.8	63.7	0.03
Appendix		75.7	76.8	73.8	0.75
Liver		23.5	18.0	29.8	–
Breast		64.8^d^	–	63.6	–
Esophagus		14.3^e^	15.0	–	–
Ovary		62.0	–	62.0	–
Prostate		33.3	33.3	–	–
Biliary^c^		15.0	50.0	–	–
Others^c^		34.4	29.5	40.5	–

a The 5-year observed survival probabilities were calculated using the life-table method.

b *P*-values were calculated using the Kaplan-Meier method. *P* values were calculated only for the top eight most common sites.

c Head and neck includes lip and oral cavity, pharynx, larynx, nasal cavity and paranasal sinuses, middle ear, and major salivary glands; Biliary includes gallbladder and extrahepatic bile duct; Others includes anus, bone, brain, cervix, intracranial gland, kidney, labia majora, mediastinum of the heart, peritoneum, pleura, retroperitoneum, skin, testis, thymus, thyroid, urinary bladder, uterus, vagina, and site undefined.

d The 5-year observed survival probability for breast cancer includes male breast cancer cases.

e The 5-year observed survival probability for esophageal cancer includes female esophageal cancer cases.

**Table 4:** Survival analysis to assess the risk of death of patients with neuroendocrine tumors, Taiwan, 1996–2009.

	Univariate				Multivariable				
		HR^a^	95% CI^a^	P		HR^b^	95% CI^b^	P	
**Primary tumor sites**									
Rectum		Referent				Referent			
Lung and bronchus		5.36	4.31–6.67	<0.0001		4.16	3.33–5.19	<0.0001	
Stomach		3.99	3.02–5.27	<0.0001		2.90	2.19–3.85	<0.0001	
Pancreas		5.04	3.80–6.68	<0.0001		5.36	4.04–7.12	<0.0001	
Colon		3.40	2.49–4.66	<0.0001		3.05	2.22–4.17	<0.0001	
Small intestine		3.51	2.57–4.79	<0.0001		2.48	1.81–3.39	<0.0001	
Head and neck^c^		3.72	2.67 –5.18	<0.0001		3.16	2.26–4.41	<0.0001	
Appendix		1.38	0.87–2.18	0.17		1.25	0.79–1.98	0.33	
Liver		7.17	4.78–10.74	<0.0001		7.28	4.86–10.93	<0.0001	
Breast		1.87	1.01–3.48	0.048		2.07	1.10–3.88	0.02	
Esophagus		8.65	5.25–14.25	<0.0001		6.60	3.99–10.90	<0.0001	
Ovary		1.84	0.86–3.96	0.12		2.45	1.13–5.28	0.02	
Prostate		5.74	2.80–11.77	<0.0001		3.18	1.55–6.55	0.002	
Biliary^c^		7.28	3.81–13.92	<0.0001		5.53	2.88–10.61	<0.0001	
Other^c^		5.05	4.03–6.31	<0.0001		5.12	4.09–6.42	<0.0001	
**Sex**									
Male		Referent				Referent			
Female		0.58	0.51–0.66	<0.0001		0.60	0.53–0.69	<0.0001	
**Age, years**									
age<40		Referent				Referent			
40< = age<50		1.13	0.86–1.50	0.38		1.12	0.85–1.48	0.43	
50 = <age<60		2.02	1.58–2.60	<0.0001		1.88	1.46–2.42	<0.0001	
60 = <age<70		2.66	2.08–3.39	<0.0001		2.39	1.87–3.06	<0.0001	
age = >70		4.32	3.43–5.43	<0.0001		3.57	2.82–4.52	<0.0001	

a Hazard ratio and 95% confidence interval were calculated using Cox proportional hazards model.

b Hazard ratio and 95% confidence interval were calculated using Cox proportional hazards model, adjusted for all of the variables in the table.

c Head and neck includes lip and oral cavity, pharynx, larynx, nasal cavity and paranasal sinuses, middle ear, and major salivary glands; Biliary includes gallbladder and extrahepatic bile duct; Others includes anus, bone, brain, cervix, intracranial gland, kidney, labia majora, mediastinum of the heart, peritoneum, pleura, retroperitoneum, skin, testis, thymus, thyroid, urinary bladder, uterus, vagina, and site undefined.

Abbreviations: CI, confidence interval; HR, hazard ratio.

## Discussion

Using the TCR data, we observed that the age-standardized incidence rate of NETs in Taiwan increased steadily from 1996 to 2001 and in a more accelerated speed since 2002. The possible reasons for this increase include the introduction of WHO classification for NETs, the improved quality of cancer registration, the increased awareness of NETs by clinicians, and the improved diagnostic technology. Despite such increase, the age-standardized incidence rate of NETs in Taiwan remained lower than those of Norway and the US. During 2000–2004, the age-standardized incidence rate of NETs was 4.06 per 100,000 in Norway, 4.92 to 5.79 per 100,000 among US Whites, and 6.82 to 7.67 per 100,000 among US Blacks [1], [2]. Our results showed that the age-standardized incidence rate of NETs in Taiwan ranged from 0.55 to 0.89 per 100,000 from 2000 to 2004. Even with the accelerated increase in the incidence rate of NETs in Taiwan since 2002, it remained lower than those of Norway and the US with the age-standardized incidence rate of NETs in Taiwan in 2008 being 1.51 per 100,000. In the US, the incidence rate of NETs varied by race, with Asian/Pacific Islanders (3.19 per 100,000) and American Indians/Alaska Natives (3.07 per 100,000) having a lower incidence rate than Whites (4.92 to 5.79 per 100,000) and African Americans (6.82 to 7.67 per 100,000) [1], [2]. Even when compared to the incidence rate of NETs among Asian/Pacific Islander in the US, the incidence rate of NETs in Taiwan is still lower. The difference in the incidence rates of NETs between races suggests the role of genetic factors, which is supported by a positive association between family history of cancer and NET risk [10], [11]. The higher incidence rate of NETs of Asian Americans compared to that of Asians in Asia suggests that perhaps environmental factors, particularly lifestyle factors, may also be important in the development of NETs. To date, information regarding the risk factors of NETs has been scarce.

Hassan et al. reported that family history of any cancer was associated with an increased risk of NETs [10], [11]. They also observed that diabetes mellitus was a significant risk factor for gastric NETs, particularly among women, although this result was based on a small number (n = 55) of gastric NET cases [10]. These findings do not appear sufficient to explain the rise in the incidence rates of NETs in the US, Norway, and Taiwan and the lower incidence rate of NETs in Taiwan compared to those in the US and Norway. Further investigations are warranted to determine the genetic and environmental risk factors of NETs. Recent studies have made progress to understand the molecular pathogenesis of NETs, including the roles of peptide receptors, receptor tyrosine kinases, and intracellular targets, such as mTOR [12]. Combining the investigation of molecular targets and the epidemiologic studies of NETs will help us identify the causes of NETs.

While there appeared to be an overall increase in the incidence rates of NETs, this increase differed by sites. In our analysis, we observed that the fastest rise in the incidence rate of NETs occurred in lung and rectum. In the US, the incidence rates of NETs increased the fastest in lung, rectum, and small intestine [2]. It is not clear why the rise in the incidence rate of NETs appeared faster for certain body sites. Investigating the changes in the incidence rate of NETs by different body sites may provide clues to the causes of NETs.

Comparing our results with those of Hauso et al. [1] and Yao et al. [2], the sites of NETs appeared to differ by race/ethnicity (Table 5). The top five NET sites in Norway were small intestine (26%), lung (21%), colon (8%), rectum (7%), and pancreas (7%) [1]. Among US Whites, the top five NET sites were lung (30% to 32%), small intestine (18%–19%), rectum (12%), colon (7%–8%), and pancreas (4%–7%) [1], [2]. Among African Americans, the top five NET sites were rectum (26% to 27%), small intestine (21% to 22%), lung (18%), colon (8%), and stomach (5% to 6%) [1], [2]. The top five NET sites among US Asians/Pacific Islanders were Rectum (41%), lung (15%), pancreas (8%), small intestine (8%) and stomach (6%) [2]. In our analysis, the top 5 NET sites in Taiwan were rectum (25%), lung (20%), stomach (7%), pancreas (6%), and colon (5%). A lower percentage of NETs in Taiwan was located in the small intestine (5%) compared to the NETs in Norway (26%) and US (Whites: 18%–19%, African Americans: 21%–22%, Asians/Pacific Islanders: 8%) [1], [2]. One may wonder whether the low percentage of NETs in the small intestine in Taiwan may be due to the clinical practice. Tumors in the small intestine, especially those located in jejunum and ileum, are difficult to identify compared to those located in duodenum or stomach, where tumor can be identified by gastroendoscopy arranged conveniently either in elective health examination or in clinical visit under the national health insurance coverage of Taiwan. However, in Yao’s report, the incidence rates of NETs in duodenum and jejunum/ileum among Asians/Pacific Islanders (0.18 and 0.09 per 100,000) were much less than those among Whites (0.15 and 0.71 per 100,000) and African Americans (0.64 and 0.88 per 100,000), suggesting that the lower percentage of small intestinal NETs in Taiwan may not have been an underestimation. In addition, a high percentage of NETs occurring in the small intestine has been reported by three other European countries, including Italy (24%) [13], Germany (22%) [14] and Sweden (35%) [15]. In studies that included only GEP-NETs, the percentages of GEP-NETs occurring in the small intestine were lower in Asian countries (<10%) [5], [16] compared to those in European countries (15–39%) [17]–[20]. In contrast to the lower percentage of NETs located in the small intestine among Taiwanese, Taiwanese has a higher percentage of rectal NETs compared to Norwegians and US Whites. Although it is possible that the difference in colonoscopy screening practice may contribute partly to this disparity, it has been shown that even within the US, the percentage of rectal NET differed by race, with Asians/Pacific Islanders having the highest percentage of rectal NET (41%) followed by American Indians/Native Americans (32%), African Americans (26%) and Whites (12%) [2]. A low percentage of rectal NET was reported by three other European (3–8%) registry-based studies [13]–[15]. In studies that included only GEP-NETs, the percentage of rectal GEP-NETs was higher in a study from Korea (48%) [16] compared to the percentages of rectal NETs in European countries (6–14%) [17], [18]. Further investigations are warranted to determine the racial/ethnic difference in the occurrence of NETs by sites.

**Table 5:** Top five most common sites of neuroendocrine tumors in Taiwan, Norway, and USA^a^.

	Taiwan		Norway		US White		US African American		US Asian/Pacific Islander	
Ranking	Site	% of allNETs	Site	% of all NETs	Site	% of all NETs	Site	% of all NETs	Site	% of all NETs
1	Rectum	25	Small intestine	26	Lung	30–32	Rectum	26–27	Rectum	41
2	Lung	20	Lung	21	Small intestine	18–19	Small intestine	21–22	Lung	15
3	Stomach	7	Colon	8	Rectum	12	Lung	18	Pancreas	8
4	Pancreas	6	Rectum	7	Colon	7–8	Colon	8	Small intestine	8
5	Colon	5	Pancreas	7	Pancreas	4–7	Stomach	5–6	Stomach	6

a References for data from Norway and US:

1 Hauso O, Gustafsson BI, Kidd M, Waldum HL, Drozdov I, et al. (2008) Neuroendocrine tumor epidemiology: contrasting Norway and.

North America. Cancer 113∶2655–2664.

2 Yao JC, Hassan M, Phan A, Dagohoy C, Leary C, et al. (2008) One hundred years after “carcinoid”: epidemiology of and prognostic.

factors for neuroendocrine tumors in 35,825 cases in the United States. J Clin Oncol 26∶3063–3072.

Abbreviations: NETs, neuroendocrine tumors.

According to our analysis, the age-standardized incidence rate of NETs was much higher among Taiwanese men compared to women with M/F incidence rate ratios ranging from 1.4 to 2. The age-standardized incidence rates of NETs were also higher in men than in women in Norway and the US, although with a smaller gap (male to female incidence rate ratio: 1.1 to 1.2) [1], [2]. Compared to the M/F case number ratios of the most common cancer subtypes by primary sites using data from the TCR [6], the M/F case number ratio of NETs appeared similar (*P*>0.05) to the M/F case number ratio of AC in rectum, stomach, colon, and small intestine but were much lower (*P*<0.0001) than that of SCC of lung or head and neck (Figure 2). The SCC of lung and head and neck share cigarette smoking as a strong risk factor [21]. In addition, the SCC of head and neck can be caused by the consumption of alcohol and betel quid [21]. The large difference in the M/F case number ratios between NETs and SCC in lung or head and neck suggests that NETs and SCC may have different risk factors. The only two studies that examined the relationship between alcohol drinking or cigarette smoking and the risk of NETs found no significant associations [5], [10]. Further investigations are required to explain the gap in the incidence rates of NETs between men and women.

The 5-year observed OS of NETs in Taiwan was comparable with those of Norway and the US for most sites except for lung and bronchus and small intestine. The 5-year OS of bronchopulmonary NET was 54%, 48% and 36% for Norwegians, US Whites, and US Blacks, respectively [1], whereas the 5-year OS of bronchopulmonary NETs was 34% in our Taiwanese population. The major subtypes of bronchopulmonary NET are typical carcinoid, atypical carcinoid, and neuroendocrine carcinoma, which includes large cell neuroendocrine carcinoma. Patients with carcinoid tumors in lung and bronchus have the best 5-year survival (78% to 97%), followed by atypical carcinoid (35% to 75%) and large cell neuroendocrine carcinoma (<50%) [22]–[28]. We suspect that the distribution of bronchopulmonary NET subtypes differs by race/ethnicity, resulting in different outcomes. There has been no large-scaled population-based data regarding the distribution of bronchopulmonary NET subtypes but two hospital-based NET series from Italy and the US showed that the percentage of typical carcinoid tumor in lung and bronchus ranged from 60% to 67% [29], [30], compared to only 32% of typical carcinoid among the bronchopulmonary NET cases in our Taiwanese population. Even with the inclusion of atypical carcinoid, only 37% of bronchopulmonary NET in Taiwan were either typical or atypical carcinoid, while 79% to 81% of bronchopulmonary NET were either typical or atypical carcinoid in Italy and the US [29], [30]. A hospital-based series from Korea reported that the percentage of carcinoid (typical and atypical) in bronchopulmonary NET was 57% [31], suggesting that Asians may have a lower percentage of bronchopulmonary NET with either typical or atypical carcinoid subtype. The worse OS of bronchopulmonary NET in Taiwan could be explained by the higher percentage of bronchopulmonary NET with the poor-prognosis subtypes. The distribution of NET subtypes in other sites also differs by countries. An article by Niederle et al. from Austria reported that the percentage of carcinoid tumor in GEP-NETs (M code: 8240), was 80%, 90%, 14%, 88%, 24%, 30% and 81% in stomach, appendix, small intestine (excluding duodenum), rectum, pancreas, colon and duodenum, respectively [17]. In our data, the percentage of carcinoid tumor was 57%, 67%, 88%, 8%, and 50% in stomach, small intestine, rectum, pancreas, and colon, respectively. The percentage of carcinoid in NETs of pancreas in our study population was only 8%, which was lower than those of NETs in the other gastroenteropancreatic sites, and that could probably explain the worse prognosis of pancreatic NETs than the other GEP-NETs.

In our study, women had a significantly better 5-year observed survival of NETs of any site than men except for appendiceal NET. In Yao’s report, women had a better survival of NETs than men regardless of stage [2]. The better survival of NETs for women compared to men was also reported by other studies [13], [14], [18]–[20]. In our analysis, this sex difference in the survival of NETs was especially prominent for bronchopulmonary and pancreatic NETs, with almost a 3-fold difference. The difference in the survival of bronchopulmonary NETs between women and men in our population may be explained by the distribution of NET subtypes. For bronchopulmonary NETs in our study population, 21% of men and 63% of women had typical carcinoid, 5% of men and 6% of women had atypical carcinoid, and 73% of men and only 32% of women had neuroendocrine carcinoma. In contrast, the large sex disparity for the survival of pancreatic NET could not be explained by the different distribution of NET subtypes because approximately 90% of both men and women with pancreatic NET had neuroendocrine carcinoma with only 8% of pancreatic NET being carcinoid. In our multivariable survival analysis, being a woman remained a favorable prognostic factor for NET survival after adjusting for site and age. Further investigations are needed to explain the sex difference in NET survival.

There are several limitations that must be considered when interpreting the results of the current analysis. The TCR does not have complete information on the grade and stage of NETs for survival analysis. The rise in the incidence rate of NETs in the current analysis could be due to several factors, including the reclassification of NETs by WHO in 2000, the improvement in the accuracy of reporting, and the increasing number of reporting hospitals. The number of NET cases may have been underestimated, particularly for benign-appearing NETs (carcinoid tumor) diagnosed prior to the first revision of WHO NET classification in 2000. However, the incidence of NETs after 2000, which was reported according the 2000 WHO NET classification, still showed a steady rise. The accuracy of cancer reporting of the TCR according to the DCO% and MV% improved from 1996 to 2008, which might have contributed to the rise in the incidence rate of NETs. However, the DCO% and MV% of the TCR did not change much between 2002 and 2008 (range of DCO%: 1.2%–3.3%; range of MV%: 87.5 to 89%) [6], during which an rise in the incidence rate of NETs still occurred. The number of cancer-reporting hospitals in Taiwan increased from 1996 to 2008. This increase reflected the establishment of new hospitals and the hospitals that expanded their facilities to 50 beds or more. The hospitals with less than 50 beds in Taiwan do not have the capacity to treat cancer patients. For these reasons, the increase in the number of cancer-reporting hospitals in Taiwan between 1996 and 2008 should not have affected our calculation of the incidence rates of NETs.

In conclusion, the current article presents the first nation-wide cancer registry-based study of NETs from Asia. Comparing with data from Norway and the US, Taiwan has a lower incidence rate of NETs. Whether this difference is due to genetics or lifestyle factors warrants further investigations. Similar to the trend of NETs occurrence in Norway and the US, the incidence rate of NETs in Taiwan has been increasing in the past decade, possibly due to clinicians’ increasing awareness of the disease and improved diagnosis but may also represent a true increase. Sparse information is available regarding the risk factors of NETs, thus, etiologic studies for NETs are needed to help devise preventive strategies. Some studies have investigated the molecular pathogenesis of NETs, including the roles of peptide receptors, receptor tyrosine kinases, and intracellular targets, such as mTOR [12]. Recently, everolimus, an mTOR inhibitor, and sunitinib, a multiple receptor tyrosine kinase inhibitor, have been proven to prolong the progression free survival of patients with GEP-NET [32]–[35]. Further studies on the molecular pathogenesis of NETs may provide us with clues on the disparity in the survival of NETs by racial/ethnic groups, by sex, and by primary sites and improve the clinical outcomes of NETs.

## Supporting Information

Table S1 ICD codes for identifying the sites of neuroendocrine tumors.
(DOC)
