# Supplementary material for: The Epidemiology of Neuroendocrine Tumors in Taiwan: A Nation-Wide Cancer Registry-Based Study
Source: PLoS One. 2013 Apr 22;8(4):e62487. doi: 10.1371/journal.pone.0062487 (PMC3632554; doi:10.1371/journal.pone.0062487)
Supplement: Table S1 — ICD codes for identifying the sites of neuroendocrine tumors. (DOC) [file pone.0062487.s001.doc]

Table S1. ICD codes for identifying the sites of neuroendocrine tumors.

| **Site** | **ICD-O-FT** | **ICD-O-3** |
| --- | --- | --- |
| **Rectum** | 1541, 1548 | C19.9, C20.9 |
| **Lung and bronchus** | 1620, 1622-1625, 1628, 1629 | C33.9, C34.0-34.3, C34.8, C34.9, C39.0, C39.8, 39.9 |
| **Stomach** | 1510-1516, 1518, 1519 | C16.0-16.6, C16.8, 16.9 |
| **Pancreas** | 1570-1574, 1578, 1579 | C25.0-25.4, C25.7-25.9 |
| **Colon** | 1530-1534, 1536-1540 | C18.0, C18.2-18.9 |
| **Small intestine** | 1520-1523, 1528, 1529, 1562 | C17.0-17.2, C17.8, 17.9, C24.1 |
| **Head and neck** |  |  |
| Lip and oral cavity | 1400, 1401, 1403-1406, 1408, 1409, 1410-1416, 1418, 1419, 1430, 1431, 1438, 1439, 1440, 1441, 1448, 1449, 1450-1456, 1458, 1459, 1498, 1499 | C00.0-00.6, C00.8-00.9, C02.0-02.3, C02.8, C02.9, C03.0, C03.1, C03.9, C04.0, C04.1, C04.8, C04.9, C05.0, C05.8, 05.9, C06.0, C06.1, C06.8, C06.9 |
| Pharynx | 1460-1469, 1480-1483, 1488, 1489, 1490, 1491, | C01.9, C02.4, C05.1, C05.2, C09.0, C09.1, C09.8, C09.9, C10.0, C10.2-10.4, C10.8, C10.9, C11.0-11.3, C11.8, C11.9, C12.9, C13.0-13.2, C13.8, C13.9 |
| Larynx | 1610-1613, 1618, 1619 | C10.1, C32.0-32.3,C32.8, C32.9 |
| Nasal cavity and  paranasal sinuses | 1470-1473, 1478, 1479, 1600, 1602-1605, 1608, 1609 | C30.0, C31.0-31.3, C31.8, C31.9 |
| Middle Ear | 1601 | C30.1 |
| Major salivary glands | 1420-1422, 1428, 1429 | C07.9, C08.0, C08.1, C08.8, C08.9 |
| **Appendix** | 1535 | C18.1 |
| **Liver** | 1550-1552 | C22.0 |
| **Breast** | 1740-1746, 1748, 1749, 1750, 1759 | C50.0-50.6, C50.8, C50.9 |
| **Esophagus** | 1500-1505, 1508, 1509 | C15.0-15.5, C15.8, C15.9, C16.0-16.2 |
| **Ovary** | 1830 | C56.9 |
| **Prostate** | 185 | C61.9 |
| **Biliary** |  |  |
| Gallbladder | 1560 | C23.9 |
| Extrahepatic bile duct | 1561, 1568, 1569 | C24.0, C24.8, C24.9 |
